# IRay therapy as an adjuvant therapy in newly diagnosed patients with neovascular age-related macular degeneration

**DOI:** 10.1038/s41433-018-0080-9

**Published:** 2018-04-17

**Authors:** Christopher Brand, Mark Arnoldussen

**Affiliations:** 1 Eye Department, Sheffield Hospitals NHS Foundation Trust, Sheffield, UK; 2 Carl Zeiss Meditec, Inc., Dublin, CA USA

**Keywords:** Macular degeneration, Radiotherapy

## Abstract

**Objectives:**

To determine the safety and efficacy at 12 months of follow-up after stereotactic radiotherapy in combination therapy with intravitreal ranibizumab injections in treatment naïve patients with neovascular age-related macular degeneration.

**Methods:**

Retrospective data analysis in patients who received stereotactic radiotherapy (IRay Therapy) during the induction phase of intravitreal ranibizumab injections and a monotherapy control group.

**Results:**

The baseline VA in the IRay and control group was 59.87 and 59.12 letters respectively. The real world visual acuity outcomes for the IRay group showed a mean gain of +3.0 letters at 12 months. The historical control group had a mean change of – 0.3 letters. The average number of injections for the IRay group and control group over 12 months was 4.45 and 5.64, respectively with three loading injections. Excluding the loading phase, the difference over 12 months was a 45.2% reduction in injections (*P* < 0.001). The number of subjects in the IRay group that didn’t require further injections following the loading phase was 45.5 vs. 24.0% control group (*P* = 0.005). The difference in mean change in central macular thickness from baseline is significant at 6 (*P* = 0.010) and 12 months (*P* < 0.01). There were no safety concerns with the IRay therapy group.

**Conclusions:**

Stereotactic radiotherapy in the induction phase of intravitreal injections of ranibizumab for treatment naïve patients with neovascular age-related macular degeneration, resulted in improved visual outcome, statistically fewer injections and statistically drier macular at 12 months, compared to historical controls treated with monotherapy intravitreal ranibizumab injections.

## Introduction

Neovascular age-related macular degeneration (nAMD) is the leading cause of blindness in the developed world [1]. In the UK, the National Institute for Health and Care Excellence (NICE) published its guidelines for the treatment of nAMD in the National Health Service (NHS) with ranibizumab in 2008 [2]. The randomized controlled trials Comparison of age-related macular degeneration Treatment Trials (CATT) [3] and Inhibit VEGF in age-related choroidal Neovascularisation (IVAN) trial [4] demonstrated that intravitreal ranibizumab and bevacizumab had nearly identical effects on visual acuity and that less than monthly or PRN dosing did not compromise vision.

Ionizing radiation has been proposed as a treatment for nAMD because it can inhibit inflammation and fibrosis and can induce regression of new blood vessels [5]. The IRay radiotherapy system (formerly Oraya Therapeutics, now Carl Zeiss Meditec) is a low-voltage, external-beam, stereotactic radiotherapy (SRT) instrument that delivers 16 Gray ionizing radiation noninvasively to nAMD lesions [6]. The system generates low-energy x-rays with precise collimation of the beam and real-time tracking of eye movement to target small areas in the eye accurately. The 90% isodose treatment zone at the macular is 4 mm with a steep decline in dose beyond this area. The purpose of this investigation was to determine if a single dose of IRay Therapy, in conjunction with intravitreal ranibizumab, could reduce the frequency of PRN injections while maintaining or improving visual acuity in newly diagnosed treatment naïve patients compared to monotherapy historical controls.

## Methods

To deliver IRay therapy, a contact lens stabilization device was coupled to the cornea using minimal suction, and eye movements were tracked via three fiducial marking retro-reflectors located on the stabilization device. Radiotherapy was delivered using 2 or 3 points of entry through the inferior pars plana. The beams overlapped on the macula to deliver the desired treatment dose (5.33 Gray per beam for 3 beams and 8 Gray per beam for 2 beams) for a total dose of 16 Gray. The dose was controlled throughout the duration of exposure; eye movements were monitored in translational *x*, *y*, *z*, and rotational axes through a multivariate algorithm that interrupted treatment if predetermined thresholds were exceeded (so called gating).

The best responder criteria in the INTREPID trial was published in the article Stereotactic Radiotherapy for wet age-related macular degeneration [7, 8]. The influence of baseline characteristics on clinical response showed at 52 weeks IRay therapy was most effective for lesions ≤4 mm in greatest linear dimension and with a macular volume greater than the median value of 7.4 mm^3^. Other features associated with a positive response to IRay therapy included pigment epithelial detachment and the absence of fibrosis.

The primary inclusion criteria for the newly diagnosed cohort of patients to receive IRay therapy was the greatest linear dimension of the active choroidal neovascular membrane (CNVM) following fundus fluorescein angiography (FFA) had to be less than 4 mm centered on the fovea, guaranteeing that the entire membrane would be exposed to the radiation beam. The active leaking CNVM was presumed to represent actively proliferating endothelial cells within the neovascular complexes. The macular volume was not part of the inclusion criteria. Additional criteria were that the best corrected visual acuity (BCVA) must be better than 6/60 and that there was no fibrosis at baseline and no permanent structural damage to the central fovea. Patients with and without pigment epithelial detachment was included in the IRay treatment cohort.

All data were recorded using a single electronic medical record (EMR) system (Medisoft Ophthalmology, Leeds, UK), which mandated collection of a standardized data set throughout the nAMD care pathway. Patients with suspected diagnosis of nAMD following history and examination, had their best corrected visual acuity documented and underwent FFA and optical coherence tomography (OCT). Patients fulfilling the inclusion criteria were fully consented. This included noting that undesired radiotherapy effects may not present themselves for several years, which could have been a factor in the decision for younger patients (youngest was 63, 7.5% under 70). Patients with diabetes mellitus were made aware of the potential increased risk of undesired radiotherapy effects.

The IRay group received an intravitreal ranibizumab injection, followed by IRay therapy within 14 days of the first injection, a second intravitreal ranibizumab injection 1 month after the first and a third intravitreal ranibizumab injection 1 month after the second.

Following the induction phase of treatment patients were reviewed and received further intravitreal ranibizumab injections on a PRN basis. At each review the patients BCVA was recorded, color fundus photograph and OCT were performed. The intravitreal ranibizumab re-treatment criteria were the presence of new hemorrhage on color fundus photograph or fundoscopy and/or the presence of new or increasing sub-retinal or intra-retinal fluid on OCT (Spectralis, Heidelberg Engineering Ltd, Germany). One hundred and thirty-three consecutive patients with CNVM secondary to nAMD that matched the inclusion criteria were recruited and treated with IRay Therapy from May 2014 to November 2015.

The size of the historical control was chosen to achieve a statistical power of 80% in determining a 0.5 injection difference with an overall alpha level of 0.05. For this, we needed in excess of 42 historical controls to make comparisons with the IRay therapy cohort. Using the eye department EMR, we designed a search query for newly diagnosed treatment naïve patients receiving intra-vitreal ranibizumab for nAMD from April 2014 to March 2015. This time period overlaps with the subjects treated with IRay therapy, ensuring identical diagnostic procedures and clinical workflow in each group. Consecutive patient hospital record codes were used to assess the patient’s FFA images and OCT scans prior to their first intravitreal ranibizumab injection. Patients qualified to be an historical control if they met the same inclusion criteria as the IRay group. The BCVA were better than 6/60 and there was no fibrosis at baseline and no permanent structural damage to the central fovea. The historical control patients had to have received 3 intravitreal ranibizumab injections a month apart in the induction phase and received further treatment as required using the same re-treatment criteria as the IRay therapy cohort. The final result from the EMR search process was 50 match historical controls which met the same inclusion criteria as the IRay therapy cohort.

Of the 133 newly diagnosed nAMD patients treated with IRay therapy, one died within the first few months into the loading phase and three passed away during the follow-up period. None of the mortalities were eye related or associated with the IRay procedure. Since the one that died before follow-up data could be acquired, 132 patient records were used for analysis of the treatment effect of IRay therapy.

Efficacy endpoints at 12 months included the mean number of PRN injections following the loading phase, the change in mean best-corrected visual acuity from baseline, and the change in mean central macular thickness (CMT) measured from OCT.

## Results

### Baseline demographics

The baseline demographics of the IRay group and the control group were similar in terms of age, gender, ethnicity, visual acuity, and lesion classification. The average age of the IRay subjects was 79.65 ± 7.10 years at the time of starting treatment, vs. 80.54 ± 7.22 years for the control group. The IRay group was composed of 62.4% female subject vs. 66.0% in the control group. The mean visual acuity at baseline was 59.87 ± 14.46 letters for the IRay group and 59.12 ± 14.27 letters for the control group. The ethnicity of both groups was primarily Caucasian (91.0% in IRay and 98% in control) with all others constituting the rest of each group. The types of lesions in the each group were similar, IRay vs. control (21.1 vs. 26.0% classic, 3.0 vs. 0.0% predominantly classic, 12.8 vs. 2.0% minimally classic, 40.6 vs. 44.0% occult, and 21.8 vs. 28.0% RAP).

### Time from first injection to IRay therapy

The average time from the first loading phase injection to the IRay treatment was 4.30 ± 3.53 days (range 2–16 days). Considering the regular workflow of the days for injection and the days for treating with IRay, subjects were primarily injected 2 or 9 days prior to IRay therapy. There was no apparent association of duration prior to IRay therapy and treatment outcomes.

### Visual acuity over 12 months

**Fig. 1:**
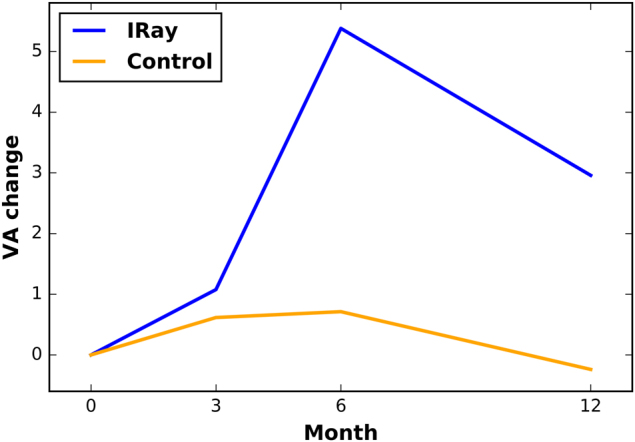
Change in visual acuity from baseline (*y*-axis) against time (*x*-axis in months) for IRay therapy and historical Control group

The baseline VA in the IRay group was 59.87 ± 14.46 letters while the control group was 59.12 ± 14.27 letters, which was not significantly different (*P* = 0.377). Over the course of 12 months of follow-up, the IRay group trended to show improved vision compared to the control, as shown in Fig. 1. While the improvement was significant at 6 months (*P* = 0.015), the trend did not maintain significance at 12 months (*P* = 0.151). The proportion of eyes in the IRay group with a visual acuity of 70 letters or more was 42% at baseline and 40% at 12 months. The corresponding percentages for the historical control group are 37% at baseline and 26% at 12 months. The proportions of eyes with 12 months follow-up in the IRay group that gained 5, 10, or 15 letters were 43, 30, and 21%, respectively, and the proportion losing 5, 10, or 15 letters were 26, 16, and 9% respectively. The proportion of eyes with 12 months follow-up in the control group that gained 5, 10, or 15 letters were 43, 26, and 23% respectively, and the proportion losing 5, 10, or 15 letters were 39, 28, and 23% respectively.

### Injections over 12 months

**Fig. 2:**
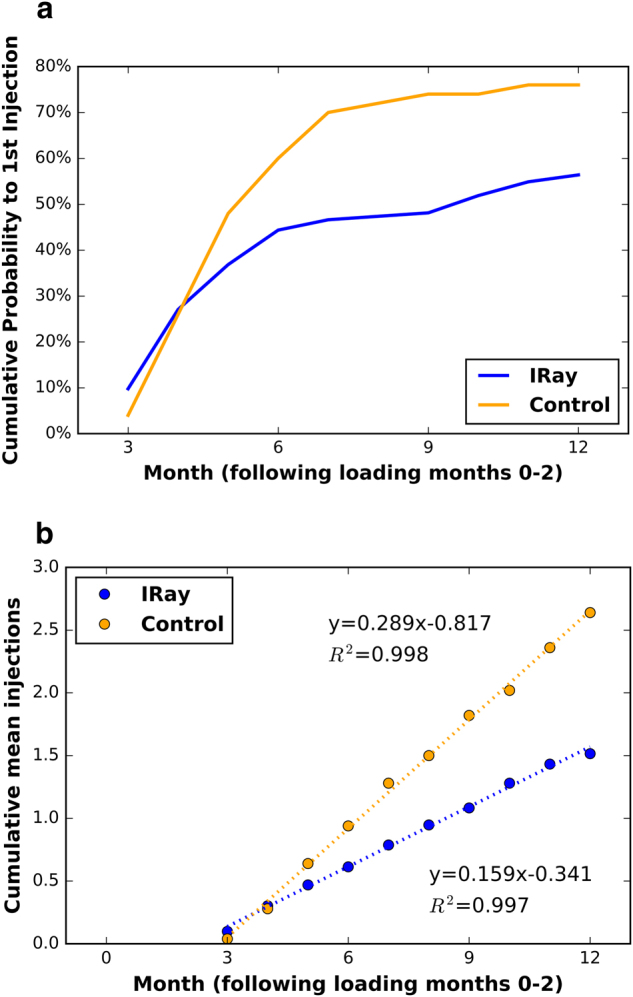
**a** Cumulative probability to first injection (*y*-axis) against time following load phase (*x*-axis in months after month 2) for IRay therapy and historical Control group. **b** Cumulative means injections (*y*-axis) against time following load phase (*x*-axis in months after month 2) for IRay therapy and historical Control group

Following the loading phase of injections administered in months 0–2, the first injection that was required based on follow-up criteria was plotted as a Kaplan–Meier cumulative probability (Fig. 2a). The difference in the cumulative probability of first injection is significant at 6 months (*P* < 0.05) and remains so thereafter (*P* < 0.01 months 7–12).

The average number of injections for the IRay group over 12 months was 4.45 ± 1.85, with three loading injections included. The average number of injections for the control group over 12 months was 5.64 ± 2.22, with three loading injections included. The monthly cumulative PRN injections excluding the loading phase are shown in Fig. 2b, and the difference at 12 months of follow-up of 1.19 injections, represents a 45.2% reduction in injections (*P* < 0.001). This significance is also reflected in the fitted linear least-squares line of the cumulative mean injections (*R*^2^ > 0.99), where the injection rate for IRay Therapy (0.159 injections/month) is nearly half the rate seen in the control group (0.289 injections/month).

**Fig. 3:**
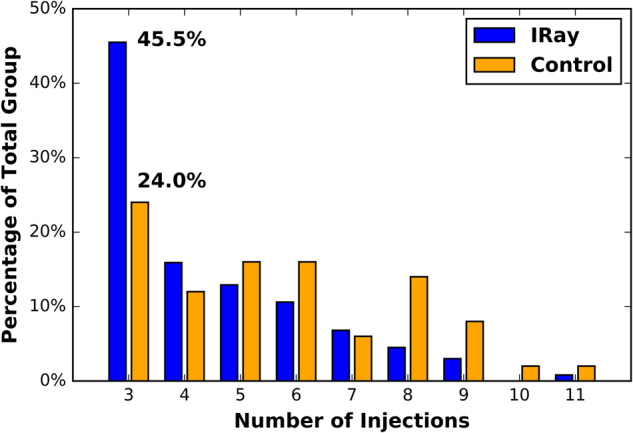
Percentage of total group (*y*-axis) against number of injections (*x*-axis) for IRay therapy and historical Control group

The distribution in the number of injections for each group over 12 months is shown in Fig. 3. With a loading of 3 injections, the number administered in each group ranged from 3 to 11 injections. The number of subjects in the IRay group that didn’t require further injections following the loading phase was nearly double that of the control group (45.5 vs. 24.0%, *P* = 0.005).

### Clinic visits over 12 months

The number of clinical visits over 12 months was lower in the IRay group compared to the control (10.13 vs. 10.60 visits, *P* = 0.023).

### Central macular thickness over 12 months

**Fig. 4:**
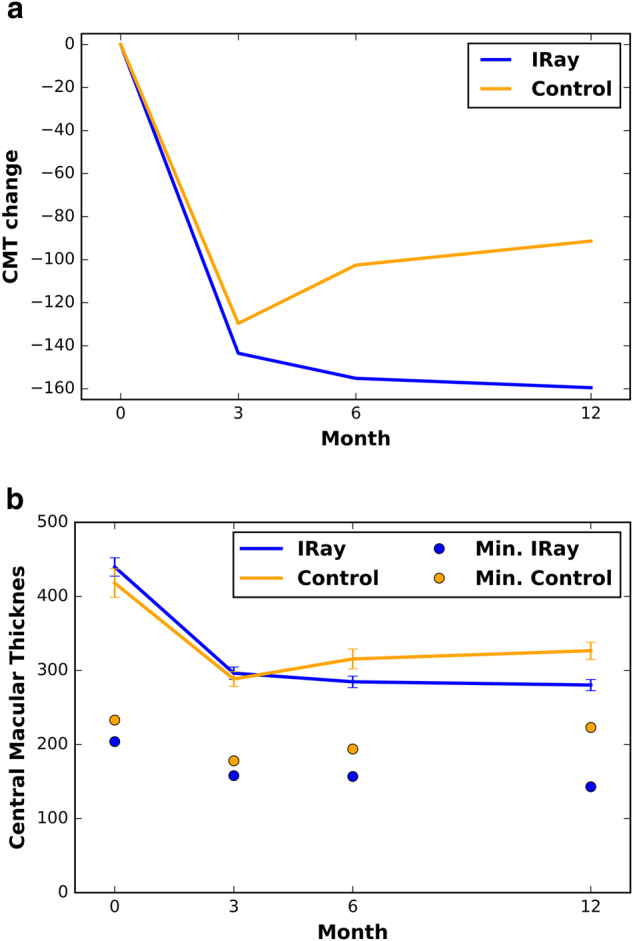
**a** Change in central macular thickness (CMT) from baseline (*y*-axis) against time (*x*-axis in months) for IRay therapy and historical control group. **b** Central macular thickness (*y*-axis) against time (*x*-axis in months) for IRay therapy and historical control group

The baseline CMT in the IRay group was 439.8 ± 143.36 micrometers while the control group was 418.04 ± 135.46 micrometers, which was not significantly different (*P* = 0.171). The difference in mean change in CMT from baseline became significant at 6 months (*P* = 0.010) and maintained significance at 12 months (*P* < 0.01), as shown in Fig. 4a. In Fig. 4b, we have shown absolute values for the CMT to clarify any concerns of foveal thinning.

## Safety

There was a slight trend in lower occurrence of fibrosis, with IRay subjects developing fibrosis in 13.6% after 12 months vs. 18.0% of the control group, but this did not achieve statistical significance (*P* = 0.219).

In the IRay group, there was one single confirmed microvascular abnormality (0.75%), with one possible occurrence of cotton wool spots that cannot be certainly claimed as an effect of radiation therapy. Side effects from radiation may take at least a year to emerge and have been mostly non-vision-affecting.

Other adverse events are listed in Table 1. In comparing the rate of occurrence in each group, none of the events achieved statistical significance.

**Table 1:** Adverse events for IRay therapy and historical control group

	IRay	Control
Adverse event	Number (%)	Number (%)
RPE changes	92 (69)	30 (60)
Atrophy	38 (29)	11 (22)
Drusen	25 (19)	6 (12)
Fibrotic scar	1 (1)	1 (2)
Sub-retinal bleed	0 (0)	1 (2)
Full-thickness macular hole	1 (1)	0 (0)
Hyper-reflective material	1 (1)	0 (0)
Increase DR CWS	1 (1)	0 (0)
ERM	1 (1)	0 (0)
PCO	1 (1)	0 (0)
Deceased	4 (3)	0 (0)

## Discussion

The real world visual acuity outcomes for the IRay therapy group showed a mean gain of +3.0 letters at 12 months. The historical control group had a mean change of – 0.3 letters. The mean letter gain has to be judged in light of the baseline visual acuity. The greater visual acuity gains are usually found in eyes with lower baseline visual acuity. The baseline visual acuity for the IRay Therapy and control group was 59.87 letters and 59.12 letters respectively. In the ranibizumab EMR UK Study Users Group the mean starting visual acuity was 55 letters, increasing to 57 letters at 1 year; a gain of +2.0 letters [9]. In the UK Outcomes with aflibercept at 1 Year, using the VIEW study protocol, the mean starting visual acuity was 53.7 letters, increasing to 58.8 letters at 1 year; a gain of +5.1 letters [10]. Of significant note in this paper is data were missing for 28% of eyes at 1 year, and it was not possible to determine the cause of loss to follow-up within the data extracted from the EMR system [10]. In these eyes, the median visual acuity when last seen was 55 letters (mean, 51.4 letters), with a wide standard deviation of 20.9 letters and 25% of patients having a visual acuity of 69 letters or better [10]. The mean 12 month visual acuity for the IRay Therapy cohort, historical control group, Ranibizimab UK EMR Study group and the UK Aflibercept Group was 59.87 letters, 58.82 letters, 57 letters and 58.8 letters respectively. This data suggests the improved vision at 12 months in the IRay cohort is in keeping with other real world published outcomes in peer review journals for patients in the UK receiving either ranibizumab after a loading phase then treated as required or aflibercept in a fixed dose regimen. A review of several real-world studies as well as meta-analyses of real-world studies; show that the quality of outcomes are based upon the absolute value of visual acuity rather than the change from baseline. The so-called “ceiling effect” has been referenced in many of these studies [9, 11, 12], representing the notion that patients with poorer vision tend to show more gain. Our historical control group falls well within the outcomes experienced by other studies in terms of 12 month VA and the number of injections during the first year (Table 2).

**Table 2:** Real-world outcomes for the treatment of neovascular age-related macular degeneration

Study group	Baseline VA	12 month VA	12 month VA Change	12 month injections	Method after loading phase
IRay + Ranibizumab					
Brand	59.9	62.8	3	4.5	PRN
Ranibizumab monotherapy					
Brand	59.1	58.8	−0.3	5.6	PRN
Lotery [14]	57.5	57.2	−0.3	6.7	Unspecified
Holz [11]	55.4	57.8	2.4	5.0	Per physician
UK EMR Users 3YR dataset [9]	55	57	2	5	PRN
UK EMR Users 1YR^*^ dataset [9]	56.6	57.4	0.8	5.7	PRN
Chong (meta) [12]	54.1	56.05	1.95	5.5	Primarily PRN
Kim (meta, T & E) [15]	53.5	59.2	5.7	6.6	T & E
Kim (meta, PRN) [15]	55.4	57.1	1.7	5.2	PRN
Aflibercept monotherapy					
Lotery [14]	58.5	58.31	−0.19	7.0	Unspecified
Talks [10]	53.7	58.8	5.1	7.0	8 week
Eleftheriadou [16]	55.9	61.3	5.4	7.3	8 week

Real world study outcomes: Baseline VA, 12 month VA, 12 month VA change, 12 month injections, protocol after induction phase, (*) denotes data interpolated from “1-year follow up” patients in UK EMR Group paper [9], (meta) denotes results from meta-analyses

Real-world outcomes for the treatment of neovascular age-related macular degeneration

Over the course of 12 months of follow-up, the IRay therapy group trended to show improved vision compared to the historical control group. While the improvement was significant at 6 months (*P* = 0.015), the trend did not maintain significance at 12 months (*P* = 0.151). The better starting visual acuity in the IRay and control groups compared to the real world UK studies mentioned probably reflects treating a smaller lesion size at baseline.

The visual acuity in real-world data is often measured with the patients distance glasses with a pinhole correction if the vision is 6/12 (70 letters) or less, rather than subjective refractions at each visit. This may underestimate the actual changes in vision. However, it may better reflect what vision patients actually experience.

The IRay therapy group and the control group received 3 loading phase injections 1 month apart. Following the loading phase, the Kaplan–Meier cumulative probability of Fig. 2 demonstrates that the effects of radiotherapy are not immediate, but rather are manifested approximately 3–4 months following treatment. The injection-reduction effect of radiotherapy achieved significance at 6 months (*P* < 0.05) and remains so thereafter (*P* < 0.01 months 7–12).

At 12 months follow-up, the number of PRN injections in the IRay therapy group was 1.45 compared with 2.64 for the control (excluding the loading phase). This difference of 1.19 injections represents a 45.2% reduction in injections (*P* < 0.001), relatively similar to prior studies. In the target patient population identified in the INTREPID random-controlled trial, IRay therapy reduced the number of PRN injections by 55%, with the IRay therapy group having 2.08 injections at 12 months compared to the monotherapy having 4.60 injections [7]. While previously treated patients tend to require more injections on average over 12 months, the relative reduction in PRN injections is similar.

The distribution in the number of injections shown in Fig. 3 shows that the number of subjects in the IRay group that didn’t require further injections following the loading phase was nearly double that of the control group (45.5 vs. 24.0%, *P* = 0.005). While the IRay group likely contained responders to anti-VEGF therapy, the additional 21.5% of patients requiring no further injection is similar to the 33% of patients in the best-responding subgroup of INTREPID and the 25% of patients in a real life study of patient’s pre-verses post-IRay therapy [8, 13].

The IRay therapy group were mandated to have 3 loading ranibizumab injections followed by a treat as required regimen with specific re-treatment criteria. This is a well-recognized treatment protocol and allowed for comparisons with historical controls and published real world data.

In the ranibizumab EMR UK Study Users Group, a gain of +2.0 letters (baseline VA 55 letters) was realized with a mean of 5.8 injections at 12 months [9]. In the UK Outcomes with Aflibercept, a gain of +5.1 letters (baseline VA 54.2 letters) was achieved with a mean of 7.0 injections at 12 months [10]. In this study, the IRay group realized a gain of +3.0 letters (baseline VA 59.87 letters) with a mean of 4.4 injections at 12 months while the historical controls experienced a loss of −0.3 letters (baseline VA 59.12 letters) with a mean of 5.6 injections. The visual acuity in each of these four groups at 12 months was: ranibizumab EMR UK Study Users Group 57.0 letters; UK Outcome with aflibercept 58.8 letters, IRay group 62.87 letters and historical control group 58.8 letters. The starting visual acuity is one of the main baseline indicators associated with better visual acuity at any time point after initiating treatment for nAMD, e.g., 12 months. The statistically significant reduction in the number of injections in the IRay group compared to the historical control group is best explained by the IRay therapy in conjunction with the intravitreal Ranibizumab injections being superior to monotherapy in closing the CNVM.

This is a real world presentation and as such there were a number of deviations from the anticipated treatment plan. Five patients (3.8%) in the IRay therapy were switched from intravitreal ranibizumab to intravitreal aflibercept due to lack of response. The switch took place after 4 intravitreal ranibizumab injections and was due to either persistent sub-retinal or intra-retinal fluid on review. Following the change in anti-VEGF agent, the treatment protocol was PRN. One patient in this group needed a total of 11 anti-VEGF injections at month 12; three had a total of 8 injections and one a total of 7 injections at month 12. One patient was switched to intravitreal aflibercept after 2 intravitreal ranibizumab injections in anticipation of the patient going on a 6 week holiday and not due to lack of response to treatment. This patient received a total of 4 anti-VEGF injections at month 12.

Two patients (1.5%) were initially started with intravitreal aflibercept, these two patients were both second eye involvement and had therapy switched from intravitreal ranibizumab to intravitreal aflibercept when their first eye was treated. These two patients had received a total of 4 and 7 aflibercept injections at month 12. Two patients (4.0%) in the historical control group were switched from ranibizumab to aflibercept, almost the same percentage switched as in the IRay group. These 2 patients average 7.5 injections each at 12 months.

There were 2 cases in the IRay therapy group who despite being mandated to receive 3 loading injections of ranibizumab, did not receive the 3 injections. One was a 91 year old, white female, with an occult CNVM, who received 1 intravitreal ranibizumab injection at baseline, IRay Therapy 2 days after the first injection, and did not receive a further injection up to the 12 month review. Baseline visual acuity was 60 letters and baseline central macular thickness was 335 microns. Visual acuity at 12 months was 60 letters and central macular thickness 282 microns. The other case was an 83 year old, white female, with a retinal angiomatous proliferative lesion, who received 2 intravitreal injections, one at baseline and one at month 1; and did not receive any further injections up to the 12 month review. This patient received IRay therapy 9 days after the first injection. The baseline visual acuity was 75 letters and central macular thickness was 272 microns. The last observation carried forward for visual acuity was 75 letters and central macular thickness 222 microns. In both cases, all reviewed OCT images were dry and fundal images did not meet the re-treatment criteria. These 2 cases raise the suspicion that it may be adequate for some treatment naïve patients to receive one intravitreal ranibizumab injection followed by IRay Therapy within 2 weeks of the first injection and subsequent treatments be given as required.

A major potential challenge in real world treatment delivery is the provision of treatment within tight timelines. This may be a particularly important reason why real-world outcomes are not as good as desired because of under treatment or delayed follow-up. The IRay therapy group shows a significant reduction in intravitreal injection burden, which may help in real world treatment delivery and vision outcomes.

The number of clinical visits over 12 months was lower in the IRay group compared to the control (10.13 vs. 10.60 visits, *P* = 0.023). Although a fewer number of clinical visits is to be welcomed, the number of clinic visits is dependent upon a number of variable factors: the response to therapy in the eye being treated or investigated, the treatment protocol, is the other eye visually impaired or visually normal, does the patient have bilateral disease with a differential treatment response in each eye. The number of subjects in the IRay group that didn’t require further injections following the loading phase was nearly double that of the control group (45.5% vs. 24.0%, *P* = 0.005). The reduction in injection demand would result in the clinician extending review intervals, resulting in a reduced number of clinical visits.

The baseline CMT in the IRay group was 439.8 ± 143.36 micrometers while the control group was 418.04 ± 135.46 micrometers, which was not significantly different (*P* = 0.171). As seen in Fig. 4a, the difference in mean change in CMT from baseline became significant at 6 months (*P* = 0.010) and maintained significance at 12 months (*P* < 0.01). This data suggests the reduction or cessation in leakage of the CNVM in the IRay therapy group compared to ranibizuamb monotherapy is evident early after treatment induction and is maintained at 12 month review. It appears combination treatment of IRay therapy and ranibizumab injections is more successful in closing the CNVM than ranibizumab monotherapy. The concern with closure of the CNVM may be a higher risk of sub-retinal fibrosis.

However, there was a slight trend in lower occurrence of fibrosis, with IRay subjects developing fibrosis in 13.6% after 12 months vs. 18.0% of the control group, but this did not achieve statistical significance (*P* = 0.219).

In the IRay group, there was one single confirmed microvascular abnormality (0.75%), with one possible occurrence of cotton wool spot that cannot be certainly claimed as an effect of radiation therapy. Side effects from radiation may take at least a year to emerge and have been mostly non-vision-affecting. Other adverse events are listed in Table 1. In comparing the rate of occurrence in each group, none of the events achieved statistical significance.

### Limitations of the study

The biggest limitation of the study is this is not a randomized controlled trial or involving multiple centers. IRay Radiotherapy System (formerly Oraya Therapeutics Newark, now Carl Zeiss Meditec) has not sponsored a randomized trial or study in treatment naïve patients with nAMD to date. The funding of IRay therapy in the NHS is not universal and the indications for when IRay therapy may be administered vary in different UK treatment centers.

### Strength of the study

This is a real world study, using the same treatment protocol amongst both the IRay therapy group and the historical controls. The data presented has not been sponsored by Carl Zeiss Meditec or a drug company. All data has been collected prospectively on an electronic medical record with all patient records being accessible.

## Summary

### What was known before

- A single 16Gy dose of IRay Therapy significantly reduces ranibizumab retreatment for patients with nAMD, with a favourable safety profile at 1 year [7].
- Whereas chronic nAMD typically results in loss of visual acuity over time, IRay Therapy is associated with relatively well-preserved VA over 1 year.

### What this study adds

- A single 16Gy dose of stereotactic radiotherapy in the induction phase of intravitreal injections of ranibizumab for treatment naive patients with nAMD, resulted in improved visual outcome, statistically fewer injections and statistically drier macular at 12 months, compared to historical controls treated with monotherapy intravitreal ranibizumab injections.
- There were no safety concerns identified in the stereotactic radiotherapy cohort related to radiation exposure.
- There are no previous publications of IRay Therapy being used in treatment naive patients with nAMD.
